# P04.15. Patient perspectives on provider communication, self-management, and alternative medicine in conventional and naturopathic diabetes care

**DOI:** 10.1186/1472-6882-12-S1-P285

**Published:** 2012-06-12

**Authors:** K Tippens, C Szybala, P Elmer, S Saha

**Affiliations:** 1 National College of Natural Medicine, Portland, USA; 2 Portland VA Medical Center, Portland, USA

## Purpose

To understand potential differences in characteristics, experiences, and perspectives between patients with diabetes who choose naturopathic or allopathic care.

## Methods

As part of an ongoing, mixed-method study of type 2 diabetes care, a survey was administered to patients of medical internists (MDs) and naturopathic physicians (NDs) from two conventional medical centers (VA and a teaching hospital), and a naturopathic teaching clinic and private naturopathic clinics in Portland, Oregon. Using previously validated scales, we measured patients’ ratings of their providers’ participatory decision-making, their providers’ communication style, their understanding of diabetes self-care and adherence to specific self-care activities (self-management), and CAM use, attitudes, and beliefs.

## Results

To date, 45 patients (26 allopathic, 19 naturopathic) have been enrolled. Patients from MD and ND clinics differed by sociodemographic factors (i.e. gender, age, income) and diabetes status (i.e. length of diagnosis, medication use). Insurance coverage and utilization also differed, for example, 19% of ND patients had an additional source of diabetes care. Patients of MDs rated more highly their providers’ communication and decision–making style. All patients had a good understanding of and adherence to diabetes self-management practices. Most patients (86.7%) rated their self-management as average or above, with more ND patients reporting “well” or “very well” compared to MD patients (63% vs. 38%, respectively). However, patients of MDs were more likely to test their blood sugar regularly compared to ND patients (88% vs. 74%, respectively), to keep a record (85% vs. 79%, respectively), and to be taking oral diabetes medications (77% vs. 63%, respectivley) or insulin (58% vs. 53%, respectively). Alternative medicine use was high among MD patients (75% reported use). Data on attitudes and beliefs about alternative medicine will be presented.

## Conclusion

Patients of MDs and NDs differed in sociodemographic factors and their ratings of provider communication and their own self-management. Further exploration of these differences is needed.

